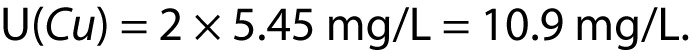# Statistical distributions commonly used in measurement uncertainty in laboratory medicine

**DOI:** 10.11613/BM.2020.010101

**Published:** 2020-02-15

**Authors:** Abdurrahman Coskun, Wytze P. Oosterhuis

**Affiliations:** 1 Department of Medical Biochemistry, Acibadem Mehmet Ali Aydınlar University, School of Medicine, Istanbul, Turkey; 2 Department of Clinical Chemistry and Hematology, Zuyderland Medical Centre, Sittard/Heerlen, The Netherlands

**Keywords:** measurement uncertainty, normal distribution, triangular distribution, uniform distribution

## Abstract

Uncertainty is an inseparable part of all types of measurement. Recently, the International Organization for Standardization (ISO) released a new standard (ISO 20914) on how to calculate measurement uncertainty (MU) in laboratory medicine. This standard can be regarded as the beginning of a new era in laboratory medicine. Measurement uncertainty comprises various components and is used to calculate the total uncertainty. All components must be expressed in standard deviation (SD) and then combined. However, the characteristics of these components are not the same; some are expressed as SD, while others are expressed as a ± b, such as the purity of the reagents. All non-SD variables must be transformed into SD, which requires a detailed knowledge of common statistical distributions used in the calculation of MU. Here, the main statistical distributions used in MU calculation are briefly summarized.

## Introduction

Uncertainty is defined as a *“non-negative parameter characterizing the dispersion of the quantity values being attributed to a measurand based on the information used”* (VIM 2.26) (*1*). Measurement uncertainty (MU) has been an important parameter in laboratory medicine for over two decades. The International Organization for Standardization (ISO) standard 15189 states that *“The laboratory shall determine the uncertainty of results, where relevant and possible”* (*2*). The ISO recently released a new standard (ISO 20914) on how to calculate the MU in laboratory medicine (*3*).

Uncertainty is considered to be an inseparable part of all types of measurements. Any measurement result without uncertainty is an abstract number. For example, a number with an absolute value, such as 10, cannot be the result of a measurement because numerically “10” means that all digits behind the last one are zero, *i.e.* 10.000…∞. Obviously, no measurement has this accuracy and therefore the number “10” itself is an abstract (hypothetical) number. In practice, we cannot measure a quantity free from uncertainty. Consequently, in metrology, the measurement results cannot be expressed as an absolute number, because of the uncertainty of this result (*4*).

In measurement procedures, we use various instruments and reagents. All data produced by instruments and reagents have a degree of uncertainty (*5*). For example, the reagents that we use for calibration will not be absolutely pure. Even if the best technology is used, a 100% purity of reagents cannot be guaranteed. Such claims are not logical and cannot be proved. Instead of an exact value, the purity should be expressed as a% ± b%, which means that the purity of the reagent is uncertain. Similar approaches can be applied for the use of glassware, pipettes, scales, and other instruments used in a laboratory setting. For example, if the volume of glassware is given as a ± b mL, such as 1000 ± 2 mL, this means that at a given temperature, the volume of the flask may vary between 998 and 1002 mL.

Measurement uncertainty can be calculated using two different approaches: Type A (bottom-up) and Type B (top-down) (*5*). In laboratory medicine, the top-down approach is preferred to calculate the MU of test results. In this method, routinely collected long-term QC data, such as internal quality control (IQC) and interlaboratory comparisons (proficiency testing (PT) or external quality assessment (EQAS)), are used to calculate MU. The calculation method is very simple, and since almost all clinical laboratories collect IQC and EQAS data, it is easy for laboratory staff to calculate the MU of measurands. However, the top-down method is not always perfect and sometimes cause incorrect or incomplete calculations (*6*). Practical calculations of MU with numerical examples using the top-down approach can be found in the literature (*7*, *8*). In contrast to the top-down approach, in the bottom-up approach, all possible sources of MU are identified and included in the calculation (*6*). This approach is more useful, especially during method development, in-house methods, and some tests that require manual interventions, such as trace elements analysis by atomic absorption spectrometry (AAS). Additionally, the new ISO document (ISO 20914) suggests a slightly different approach from the bottom up and the top down as described in Eurachem and EP29. This manuscript is appropriate also for the new ISO standard, which mainly considers the normal distribution and the “top down” approach.

**Figure 1:**
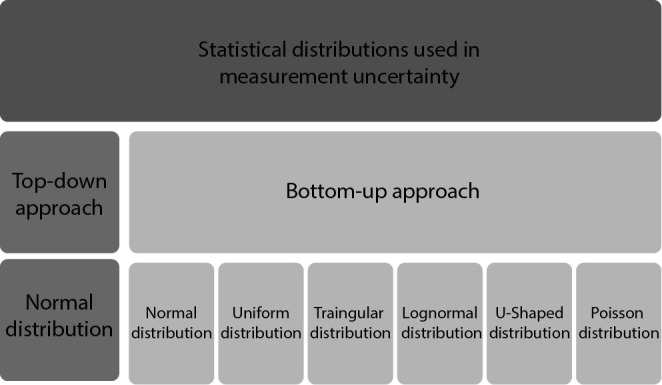
Statistical distributions used in measurement uncertainty (MU) calculation. Top-down approach is simple and preferred in MU calculation of tests analysed by auto-analysers. Bottom-up approach is preferred in methods, which require manual interventions such as trace element analysis by AAS/ICP-MS and various analytes by HPLC, LC-MS/MS, GC-MS and in-house methods and so on.

In both the top-down and bottom-up approaches, uncertainty is expressed in terms of standard deviation (SD). However, the characteristic of components of total uncertainty may be different; some of them are expressed as SD, while others are expressed as a ± b, such as the purity of the reagents. Therefore, all non-SD variables must be transformed into SD, which required a detailed understanding of common statistical distributions used in the calculation of MU. In statistics, although there are various distribution types, only a few are sufficient to calculate the MU in laboratory medicine (Figure 1). Here, we briefly summarize the main statistical distributions used to calculate MU using both the top-down and bottom-up approaches. The combination of the sources of uncertainty to obtain the total MU in laboratory medicine is also discussed.

## Probability distributions

In laboratory practice usually we are interested in different measurement systems, experiment protocols, dataset *etc.* Although we use the same sample, method, instrument and reagents the results of even repeated measurements are not always the same. When these data are examined visually on a graph, it can be seen that they exhibit a distribution. In other words, there are probabilities rather than the same values. In order to evaluate the data correctly, we have to know the characteristics of probability distribution.

In statistics, a probability distribution is a mathematical function that provides all of the possible outcomes of a random variable with their corresponding probabilities. Various probability distributions are used in statistics and they can be classified into two main groups: continuous and discrete distributions (*9*). Some of these distributions are listed in Table 1. Distributions are represented by three main parameters: mean, variance and standard deviation. These parameters are given for each distribution discussed in this paper where appropriate.

**Table 1:** Probability distributions commonly used in statistics

**Continuous distributions**	**Discrete distributions**
Normal distribution*	Binomial distribution
Uniform distribution*	Negative binomial distribution
Triangular distribution*	Beta binomial distribution
Lognormal distribution*	Poisson distribution*
U distribution*	Geometric distribution
Chi-Square distribution	Hypergeometric distribution
Student’s t distribution	Bernoulli distribution
Beta distribution	Discrete uniform distribution
F distribution	Logarithmic series distribution
Exponential distribution	Zeta distribution
*Discussed in the paper.	

## Statistical distributions used in top-down approach

In the top-down approach, we use collected QC data to calculate the MU of the measurands. Therefore, the normal distribution is the main type of distribution used in the top-down approach (Figure 1).

### Normal distribution

**Figure 2:**
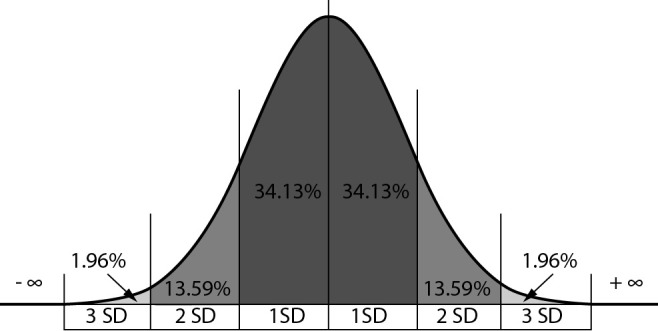
Normal distribution curve is the main distribution used in measurement uncertainty calculation of both top-down and bottom-up approaches. The total area under the normal distribution curve is equal to 1. The area under the curve between consecutive SDs exponentially decreases as it moves away from the center of the curve. SD – standard deviation.

The normal distribution is the most frequently used distribution type in laboratory medicine (Figure 2). Due to the contributions made by the famous mathematician Friedrich Gauss, it is also known as the Gaussian distribution. The result of repeated measurements, such as imprecision studies, generally corresponds to the normal distribution. The mathematical expression of the normal distribution is as follows:
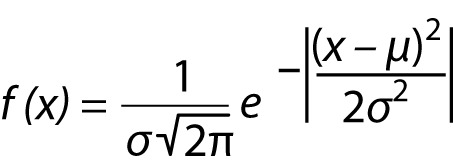

where *f*(*x*) is the normal distribution function, *σ* is the SD, *μ* is the mean, and *x* is a variable.

As shown in Eq (*1*) the mathematics of a normal distribution is extremely complex and prone to serious errors (*10*-*13*). The graph of a normal distribution is symmetric around the mean and the tails of the distributions on the left and right sides gradually approach, but never intersect, the x-axis, *i.e.* they are asymptotic to the x-axis (Figure 2). The total area under the curve (AUC) is equal to 1. The AUC between the upper and lower limits increases by moving these limits away from the mean. From this, we can make the following approximations (Figure 2):

68.3% of AUC (or results) is encompassed within the mean ± 1 SD;
95.5% of AUC (or results) is encompassed within the mean ± 2 SD;
99.7% of AUC (or results) is encompassed within the mean ± 3 SD.

The mean, variance and SD of normal distribution can be calculated using the equations given below:


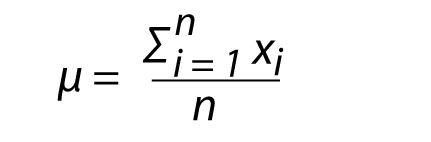



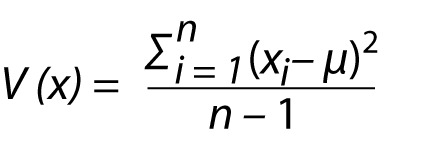



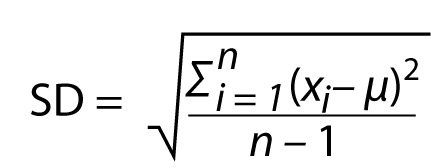


The SD of normal distribution is also the standard uncertainty of the measurements. Before using a normal distribution, we should ensure that our data conforms to this normal distribution. We can use normality tests, such as the Kolmogorov-Smirnov and/or Saphiro Wilk tests, to verify the normality of data. The probability of repeated measurement results being distributed around the mean is not the same everywhere. Thus, the dispersion of the data around the mean is not homogenous. The data is most likely to be close to the average, but exponentially decrease towards the tails (Figure 2). Repeated measurement results may correspond to a normal distribution; however, in the laboratory, many datasets may show a different distribution. We therefore need additional distribution types to cover all of the data produced in laboratory medicine.

## Statistical distributions used in the bottom-up approach

**Figure 3:**
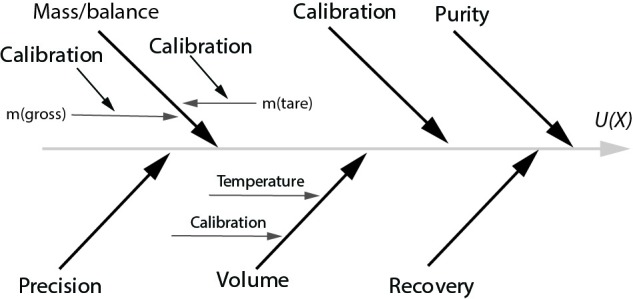
In bottom-up approach, the sources of measurement uncertainty can be illustrated by fishbone diagram. The head of the diagram shows the cumulative uncertainty, wherein the components of uncertainty are the bones of the diagram.

The bottom-up approach is not the method routinely used in automated systems in laboratory medicine. However, this approach is preferred in methods which need manual interventions, such as trace element analysis by atomic absorption spectroscopy (AAS) and inductively coupled plasma mass spectrometry (ICP-MS), as well as various analyses by high performance liquid chromatography (HPLC), liquid chromatography-tandem mass spectrometry (LC-MS/MS), gas chromatography–mass spectrometry (GC-MS), and in-house methods (*14*, *15*). In the bottom-up approach, all possible sources of MU are identified and included in the calculation. The fishbone diagram in Figure 3 shows the sources of MU. The head of the diagram shows the cumulative uncertainty, wherein the components of uncertainty are the bones of the diagram. Figure 3 shows the various sources contributing to the uncertainty of the test results. These sources form a heterogeneous group, such as imprecision, recovery, volumetric measurements, and weighing of chemicals. The characteristics of these components are not the same. For example, the imprecision is expressed as SD, but the volume of glassware is given as a ± b, such as 1000 ± 1 mL. Similarly, the purity of a chemical might be expressed as c%, such as 99.5%. However, in some cases, the manufacturers may not have provided additional information. In this case, we cannot say that the SD of the chemical is 0.5 and/or the SD of glassware is 1. Since the volume of the glassware is not reported as the mean ± SD, it is reported as a ± b, where b is not the SD. A similar approach is correct for use in chemicals. From these components, we can say that the characteristic of the data used in the calculation of MU in the bottom-up approach is frequently heterogeneous. In a statistical sense, the distribution type of these data is not the same. However, the total uncertainty is calculated by combining all these components using the Gaussian approach (taking the square root of the sum of squares of the component uncertainty). To do this, all the components must be expressed in SD or relative standard deviation (RSD); otherwise, the calculated results will be incorrect. To overcome this problem, as we mentioned previously, all non-SD variables should be transformed into SD, which required a detailed understanding and prior experience with data characteristics and the common statistical distributions used in MU calculation. The common types of distribution used in the bottom-up approach are listed below.

### Normal distribution

A detailed explanation of the normal distribution is provided in the “top-down” approach section above.

### Uniform distribution

The uniform distribution is an inseparable part of MU calculations, particularly in the bottom-up approach. As is shown in Figure 3, the uncertainty of some sources contributing to the total uncertainty is not represented as SD. In this case, the uniform distribution can be used to calculate the total uncertainty. For example, if the volume of a flask is given as 1000 ± 4 mL, and if there is no additional information, this indicates that the probability of the volume being 1000 mL, 998 mL, 1004 mL, or 1002 mL is the same. Therefore, the probability of the volume being any value between 996 and 1004 mL is the same. Statistically, this is a uniform distribution. In this case, we cannot say that the SD is 4. Because the volume of glassware is not reported as mean ± SD, it is reported as a ± b, and here, *b* is not expressed as the SD. A similar approach applies to the composition of chemicals.

**Figure 4:**
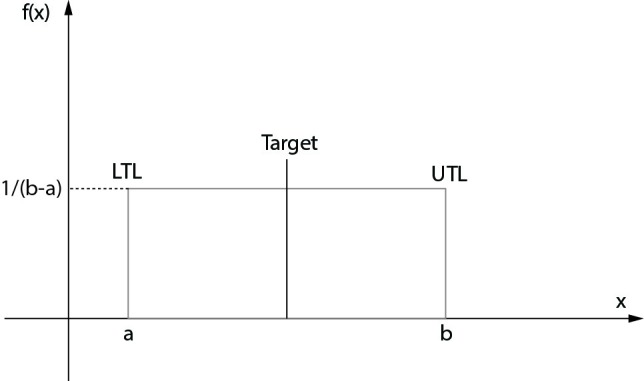
Uniform (rectangular) distribution. In contrast to other distributions, the probability density function of uniform distribution is constant between upper (UTL) and lower limits (LTL).

A uniform distribution is a continuous probability distribution with a probability density function shaped like a rectangle. It is the simplest form of continuous probability distributions, due to its shape; it is also known as a rectangular distribution (Figure 4). In contrast to the normal distribution, the mathematics of the uniform distribution is very simple:
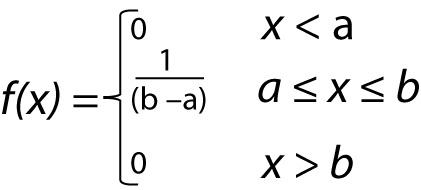

where *f*(*x*) is the uniform distribution function, *a* is the lower limit, *b* is the higher limit, and *x* is a variable (Figure 4).

As shown in Eq. 5, the probability density function is constant between *a* and *b*. The uniform distribution is a very practical and common distribution type in laboratory practice. When the uncertainty of glassware, chemicals, and weighing is included in the total uncertainty, calculations related to the uniform distribution are required. Similar to all other probability distributions, the AUC of the uniform distribution is 1. The mean, variance, and SD, respectively, of the uniform distribution are as follows (*16*):


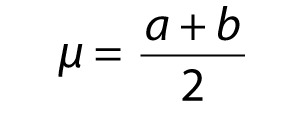



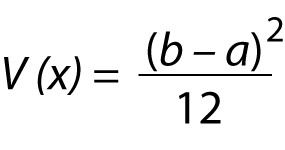



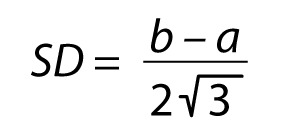


If the distribution is centered at zero with the endpoints -a and a then the equation of SD will be simplified as follow:


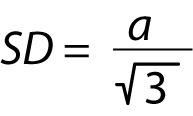


In the example given above the volume of the flask is given as 1000 ± 4 mL and the standard uncertainty would be 4/3^1/2^ = 2.31.

In addition to glassware and reagent purity a typical example of the uniform distribution is the uncertainty of age used in calculating the estimated glomerular filtration rate (eGFR) (*17*). Estimated glomerular filtration rate can be derived from serum creatinine and age of patient, as given below:


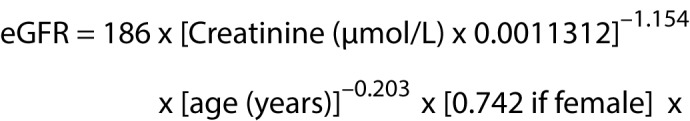


In this equation, age is given as an integer number of years and can be expressed as age ± 0.5. It follows uniform distribution and therefore the standard uncertainty of age would be 0.5/3^1/2^ = 0.29.

### Triangular distribution

**Figure 5:**
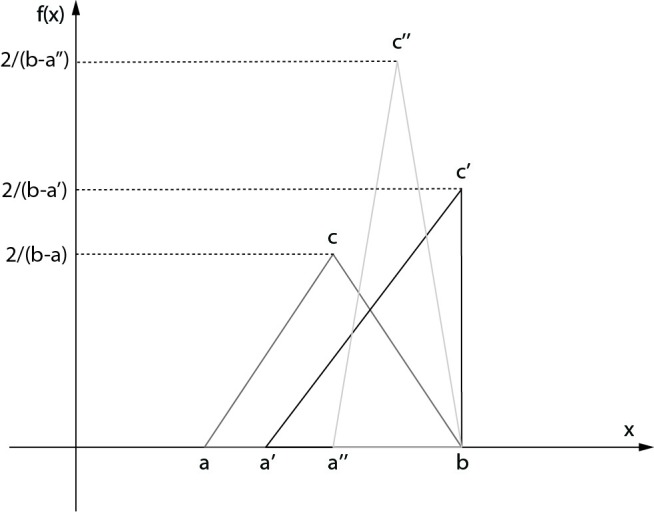
Triangular distribution. In contrast to uniform distribution, the variables are not distributed uniformly around the mean. They have a minimum (lower limit, a, a’, a’’), maximum (upper limit, b) and a peak (c, c’, c’’) value. The variables in triangular distribution may be symmetrically or asymmetrically distributed around the mean.

The triangular distribution is a continuous probability distribution with a probability density function shaped like a triangle (Figure 5). In contrast to the uniform distribution, the variables in a triangular distribution have a central tendency, and the variables are not distributed uniformly around the mean (a detailed review on triangular distribution can be found in Kotz *et al.*) (*18*). This distribution has a minimum (lower limit), maximum (upper limit), and a peak value. These makes it very easy to estimate the distribution from data. For example, if the volume of a flask is given as a ± b, such as 1000 ± 5 mL, and if we know that there is a central tendency, *i.e.* the probability of the volume being 1000 mL is more likely than 995 mL or 1005 mL, in this case, it is better to use a triangular distribution than uniform distribution.

The upper and lower limits protect the distributions from unwanted extreme values, such that the triangular distribution is very useful. In contrast to the triangular and rectangular distributions, the normal distribution has no upper or lower limits. It is expressed between –∞ and +∞, and therefore, at least theoretically, extreme values may be present within a normal distribution. Like all other probability distributions, the AUC of the triangular distribution is 1, and similar to uniform distribution, the mathematics of triangular distribution is very simple:
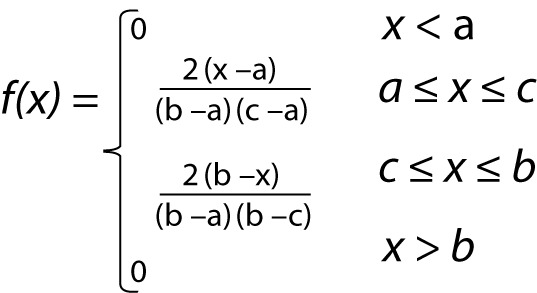
where *f*(*x*) is the triangular distribution function, *a* is the lower limit, *b* is the higher limit, *c* is the peak value, and *x* is a variable (Figure 5). As shown in Figure 5, the distribution may be symmetric around the mean or not.

If we assume that the triangular distribution is symmetric, then we can calculate the mean, variance and SD of the triangular distribution as follows (*16*).


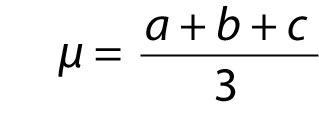



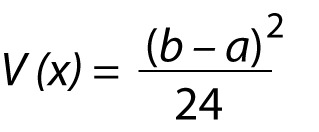



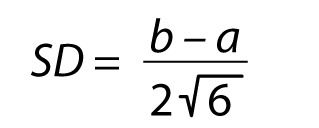


If the distribution is centered at zero with the endpoints -a and a then the equation of SD will be simplified as follow:


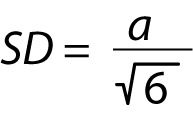


The difference between triangular and rectangular distribution has been widely questioned. How do we decide which one to use in practice? It is recommended to use triangular distribution if there are reasons to expect values near the centre than the bounds. In practice, it is rational to expect measurement results near the mean rather than extremes (*5*). Therefore, in laboratory practice if we have doubt as to which one to use, it is better to prefer triangular distribution. In the example given above the volume of the flask is given as 1000 ± 5 mL and the standard uncertainty would be 5/6^1/2^ = 2.04.

### Lognormal distribution

In statistics, if the dataset is not normally distributed, we can use some transformation techniques to obtain a normally distributed dataset. However, these transformations do not guarantee that the transformed data will be normally distributed. Frequently, log transformation is the preferred method because, even if the variables are very different, such as 10, 100 and 1000, their logarithms (ln) are 2.3, 4.6 and 6.9 respectively. If the dataset is not normally distributed, the logarithm of dataset may be normally distributed.

The lognormal distribution is a continuous probability distribution of variables whose logarithm has a normal distribution. Thus, the ln(x) is normally distributed. The mathematics of a lognormal distribution is very similar to the mathematics of a normal distribution, as follows:
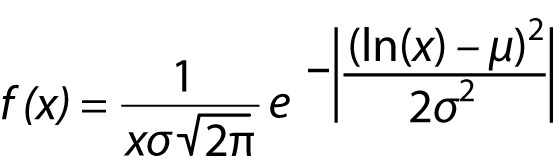
where *x* is a random variable, and *μ* and *σ* are (theoretical values) the median and SD of ln(x), respectively.

The lognormal distribution has a wide variety of applications in laboratory medicine; however, it is rarely used for calculating uncertainty. Although it is commonly used in uncertainty calculation in mechanical metrology, this is not the cases for laboratory medicine.

### U distribution

**Figure 6:**
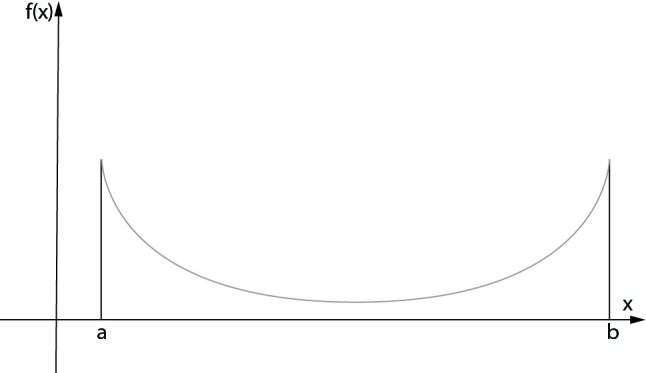
U Distribution. The probability of measured results at the upper (b) or lower (a) limits is higher than the centre.

The statistics of continuous data is usually comprised of a centre (*e.g.,* mean and target), with the data distributed around this centre. The shapes of data distributions specify the type of distributions including normal, uniform, triangular, and lognormal. However, this central tendency is not always the case. Events can occur at the extremes of the ranges rather than the centre of the distribution. Therefore, the probability of measured results at the upper or lower limits is higher than the centre. In this case, the distribution of the measured data forms the letter “U,” which represents the higher frequency of data at the upper and lower limits (Figure 6). For example, if we use a temperature controller to adjust the temperature of the laboratory between 23–25 ºC, the controller system will activate or deactivate the heating system at 23 and 25 ºC, and not at the centre (24 ºC).

The SD of a U distribution is as follows:


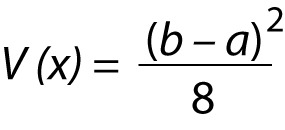



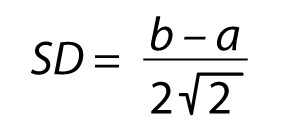


If the distribution is centered at zero with the endpoints -a and a then the equation of SD will be simplified as follow:


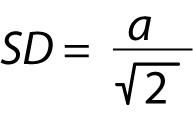


In the example given above, the standard uncertainty of heating system at the edge would be 1/2^1/2^ = 0.71.

Similar to the lognormal distribution, the U distribution is frequently used by physicists and chemists, rather than in laboratory medicine, and this type of distribution is not needed in the same way as the normal and uniform (or triangular) distributions are.

### Poisson distribution

In laboratory medicine, we do not always measure the concentration of the analytes. Sometimes the specific cell types are counted, even though these cells may be rarely seen. One example is the number of particular types of cells *per* volume of urine or *per* amount of tissue samples. In these situations, it is accepted that the counted cells are randomly distributed in the samples, where the rarely seen cells (< 10%) conform to a Poisson distribution rather than a normal distribution (*3*).

Similar to the lognormal and U distributions, Poisson distribution is also frequently used by physicists.

As shown below, in comparison to the normal distribution, the mathematics of the Poisson distribution is simple:
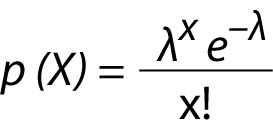
where *λ* is the mean of data and *x* is a variable, such as 1, 2, 3, and so on. It is very interesting that the variance of the count is equal to the count. In this case, the SD is equal to the square root of the count:


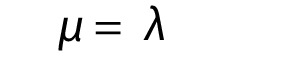



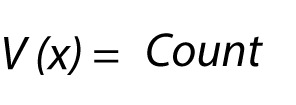



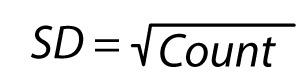


Since the MU calculation uses the SD, in the Poisson distribution we do not need a complex calculation to obtain the SD. For example if the mean number of white blood cells (WBC) counted in urine is 4 *per* high power field (wbc/hpf), the SD (standard uncertainty) of WBC in urine is the square root of the count, *i.e*. 4^1/2^ = 2 wbc/hpf.

### Combining of variances to calculate the combined standard uncertainty

In laboratory medicine, uncertainty can be considered into two main categories: analytical and diagnostic. Diagnostic uncertainty arises from various sources including analytical uncertainty and biological variation. Analytical uncertainty arises particularly from analytical and pre-analytical factors (*19*). If MU has more than one component, we must combine the uncertainty of these components to obtain the combined standard uncertainty. Mathematically, the variances (SD^2^) of two subgroups can be combined using the following equation:


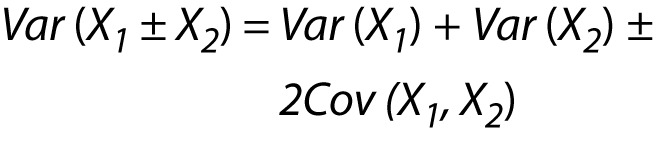



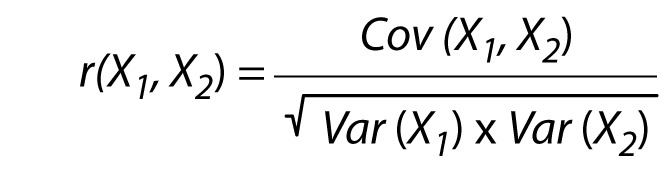


where *r* is the coefficient of correlation, *Cov* is the covariance, *X_1_* and *X_2_* are the variables. Combining Eq. 24 and Eq. 25 will yield the following equation:
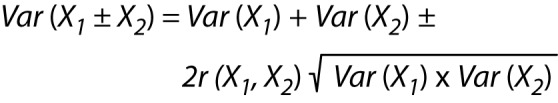
where the subgroups (*X_1_* and *X_2_*) are independent and therefore *r* is zero, whereby equation (XI) can be simplified as follows:


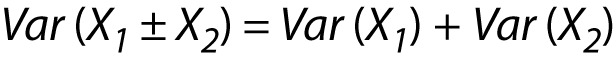


This approach can be applied to more than 2 variances, which enables calculating the total variances of various subgroups by taking only the sum of the variances.

In this approach, the combined standard uncertainty (U_CS_) is calculated using the uncertainty of each component as follows:


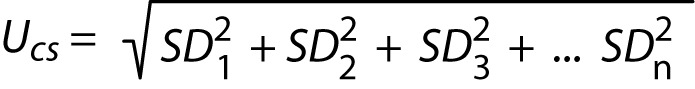


Combined standard uncertainty is not the final step and we have to calculate the expanded uncertainty *(U)* which is also requested by ISO 15189. To calculate the expanded uncertainty, we have to multiply U_CS_ by a coverage factor (*k*). From the equation of normal distribution we know that 95.5% of results is encompassed within the mean ± 2SD and therefore a value of 2 is preferred as the coverage factor.


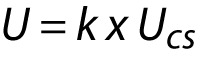


## Conclusion

Recently, the ISO released a new standard on how to calculate the MU in laboratory medicine (*3*). This standard will accelerate and facilitate the implementation of MU calculation in daily practice. The most common approach in clinical chemistry to estimate the MU is the top-down method, where the MU is derived from the available QC results. However, in some cases, such as in-house methods and analyses that involve manual interventions (*e.g.*, HPLC, AAS, ICP-MS, and LC-MS/MS), the bottom-up approach is preferred (*14*, *15*). In both the top-down and bottom-up approaches, to calculate the MU correctly, we need to know the statistical distribution of components that contribute to the MU. Contrary to popular belief, calculating MU is simple, where the use of normal and uniform (or triangular) distributions is sufficient to perform this calculation (see Appendix).

## Appendix

### Using different distribution types to calculate measurement uncertainty, a practical example

#### Preparation of a calibration standard for copper measured by AAS

The purpose of this example is not to calculate the uncertainty of the entire measurement procedure of copper by AAS, but rather to show how to apply different distribution types to the components of total uncertainty. Although this simple example does not represent the entire measurement procedure of copper, the preparation of the calibration standards is an inseparable part of the in-house methods, particularly for the measurement of trace elements by AAS.

In this example, the measurand is the concentration of copper standard solution prepared for the calibration of AAS. The concentration depends on the purity of the copper obtained from the manufacturer, the weighing of the copper, and the volume of the liquid used to dissolve the copper. The following equation can be used to calculate the concentration of copper:
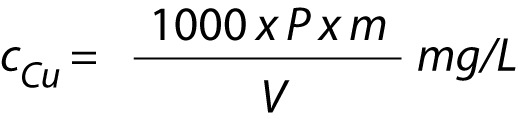
where *c_Cu_* is the concentration of copper calibration standard, *P* is the purity of copper provided by the manufacturer, *m* is the mass of the weighed copper (mg), and *V* is volume of the liquid used to prepare the calibration standard (mL).

The procedure of the preparation of the calibration standard has two steps:

Step 1. Weigh the copper.
Step 2: Dissolve and dilute.

**Figure 7:**
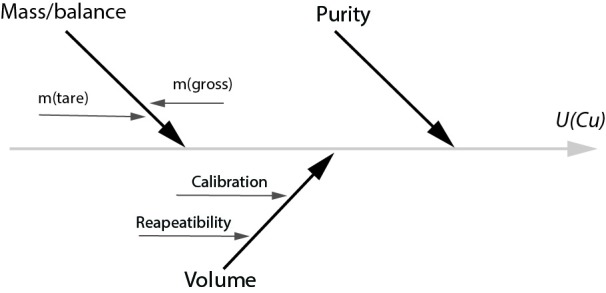
Fishbone diagram of uncertainty sources of calibration standard used to measure copper by AAS.

As shown in Figure 7, in this example, there are three main sources of uncertainty: purity (P), mass/balance, and volume.

### Purity

**Figure 8:**
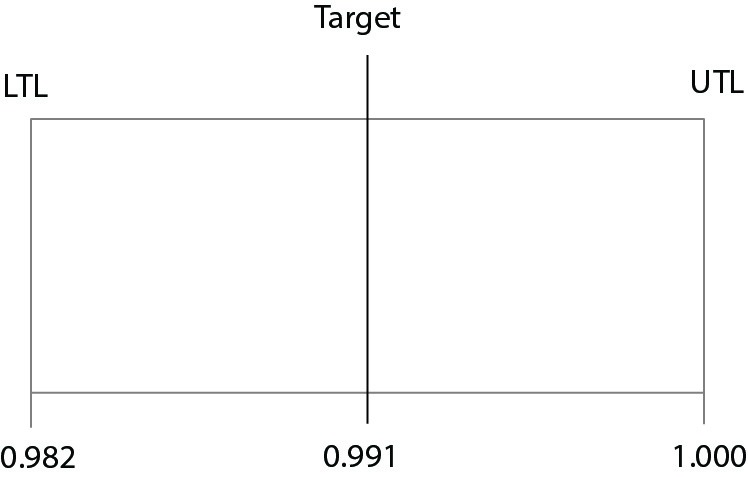
The distribution (rectangular) of the purity (P) of reagents used in calibration of copper measured by AAS. P is the same between 0.982 and 1.000.

No chemicals are 100% pure. The purity (P) of a chemical is expressed as a percentage, a ± b%. If the purity provided by the supplier is 99.10 ± 0.90%, in this case, P is 0.991 ± 0.009. If there is no additional information provided by the manufacturer regarding the uncertainty of purity, **a rectangular (uniform) distribution** can be assumed (Figure 8). In this case, equation (V) can be used to obtain the standard uncertainty as follows:


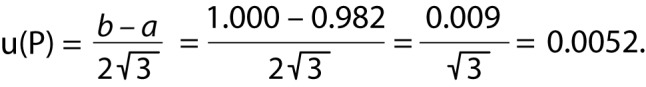


### Mass/balance

To prepare a solution, we have to weigh the chemicals. There are 3 main uncertainty sources related to weighing: repeatability, readability (the resolution of the scales used), and calibration function. Detailed calculations of the uncertainty related to mass can be very complex, such that, for the sake of simplicity, the data provided by the manufacturer in the calibration certificate should be used. If the standard uncertainty value is 0.01 mg, this data can be used directly. In a calibration certificate, if the uncertainty is expressed as SD, it can be assumed to be a **normal distribution.** As shown in Figure 7, due to tared weighing, the uncertainty related to weighing should be obtained twice.


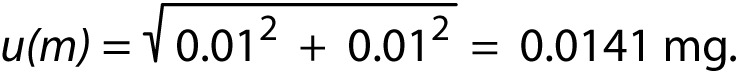


### Volume

Volume is an important source of uncertainty and has three main influences: repeatability, calibration, and temperature. If the temperature of the laboratory is regulated strictly within a given interval, then the uncertainty related to the temperature can be omitted. In this case, we have to calculate the uncertainty related to the calibration and repeatability of the flask.

- Calibration: If we use a 100 mL flask and if the volume of the flask is given as 100 ± 0.2 mL by the manufacturer, the standard uncertainty can be calculated using equation (V). If there is no additional information, we can assume the **rectangular distribution**.
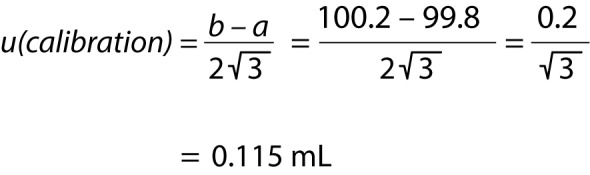

- Repeatability: The filling of the flask has a variation, whereby the uncertainty related to filling can be estimated by repeatability experiments. The 100 mL flask has to be filled and weighed at least 10 times. The repeatability experiments are expected to be **normally distributed.** If the SD obtained from the fill and weigh experiment is 0.1 mL, this data can be used directly as the standard uncertainty.

To obtain the uncertainty related to the volume, we have to combine the uncertainty calculated from calibration and the repeatability using Gaussian approach as follows:


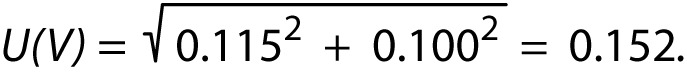


### Calculating the combined standard uncertainty

Now that we have obtained the standard uncertainty of purity, mass, and volume (Table I), all these components must be expressed as the SD (normal distribution). Thus, we have to combine these components to obtain a combined standard uncertainty.

**Table I:** The component of uncertainty of the calibration standard for copper measured by atomic absorption spectrometry

**Components**	**Value *(x)***	**Standard uncertainty *u(x)***	**Relative standard uncertainty *U(x)/x***
Volume	100 mL	0.152	0.00152
Mass	100 mg	0.0141	0.00014
Purity	0.991	0.0052	0.00525

The combined standard uncertainty is calculated for specific concentrations. Therefore, in the first step, the concentration of the calibration standard should be calculated.

Purity: 99.1%
Volume: 100 mL
Mass: 100 mg
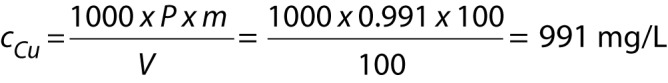

The uncertainty values for the purity, volume, and mass can be combined using Gaussian approach as follows:


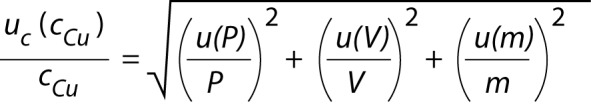



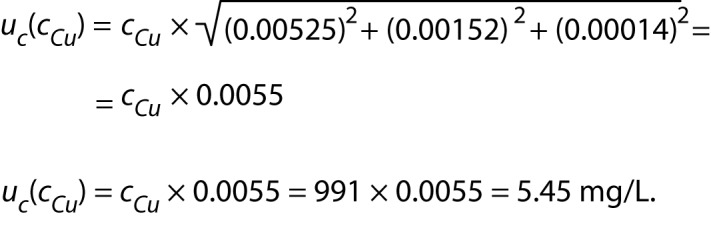


In the final step, we have to calculate the expended uncertainty. To calculate the expended uncertainty, we have to multiply combined standard uncertainty by a coverage factor (*k*). Usually, a value of 2 is preferred as the coverage factor.